# Meta-analysis of the curative effect of sacral nerve magnetic stimulation on neurogenic bladder after spinal cord injury

**DOI:** 10.1097/MD.0000000000040150

**Published:** 2024-10-18

**Authors:** Xingxing Yu, Jianye Chen, Mingda Liu, Yunfei Li, Yuyan Jia, Hongsheng Zhan, Xukai Wang

**Affiliations:** a College of Traditional Chinese Medicine, Changchun University of Chinese Medicine, Changchun, China; b The Affiliated Hospital of Changchun University of Chinese Medicine, Changchun, China; c Shi’s Traumatology Medical Center, Shuguang Hospital Affiliated to Shanghai University of Traditional Chinese Medicine, Shanghai, China.

**Keywords:** functional magnetic stimulation, meta-analysis, neurogenic bladder, sacral nerve, spinal cord injury, systematic evaluation

## Abstract

**Background:**

The curative effect of sacral nerve magnetic stimulation on the neurogenic bladder (NB) after spinal cord injury (SCI) is unclear. This study made a meta-analysis of the curative effect of sacral nerve magnetic stimulation on the NB after SCI and put forward a theoretical basis for the clinical treatment of this disease.

**Methods:**

The databases of PubMed, Embase, Cochrane Library, Web of Science, China National Knowledge Infrastructure, WanFang Data, VIP, and CBM were searched by computer, and the randomized controlled trials of sacral nerve magnetic stimulation in the treatment of NB after SCI were collected. The retrieval time was from the establishment of the database to September 25, 2023. Meta-analysis was performed with Stata 15.0 software.

**Results:**

This study finally included 10 articles, including 537 patients. Meta-analysis shows that the sacral nerve magnetic stimulation treatment group can effectively improve urodynamics: the maximum bladder capacity increases (mean difference [MD], 40.76 [95% confidence interval (CI), 33.24–48.28]; *P* ˂ .05), the bladder capacity increases at the beginning of urination (MD, 12.82 [95% CI, 11.02–14.62]; *P* ˂ .05), and the residual urine volume decreases (MD, −38.08 [95% CI, −51.36 to −24.79]; *P*˂.05). In the aspect of urination diary, the sacral nerve magnetic stimulation treatment group also has advantages compared with the conventional treatment group: the increase in single urine volume (MD, 22.49 [95% CI, 18.68–26.30]; *P*˂.05), the maximum urine volume (MD, 32.84 [95% CI, 22.37–43.30]; *P*˂.05), and the decrease in urination times (MD, −2.23 [95% CI, −3.13 to −1.33]; *P*˂.05). After the treatment of sacral nerve magnetic stimulation, the quality of life of patients can be improved: quality-of-life score (MD, −0.62 [95% CI, −0.91 to −0.34]; *P*˂.05).

**Conclusion:**

Combined with functional magnetic stimulation of the sacral nerve, routine treatment is superior to routine treatment in improving the clinical symptoms of patients with NB after SCI.

## 1. Introduction

Neurogenic bladder (NB) is also called neurogenic lower urinary tract dysfunction. Its pathology is dysfunction of urine storage and/or urination caused by a disorder of the neural control mechanism. Its specific clinical features are frequent urination, urgency, urinary incontinence, nocturia, dysuria, and incomplete bladder emptying.^[1]^ Spinal cord injury (SCI) is one of the main causes of NB.^[2]^ Trauma, tumor, or vascular diseases can cause SCI, and about 70%~84% of patients with SCI can develop NB.^[3]^ NB can cause urinary tract infection and kidney damage and increase the rate of rehospitalization, which seriously affects the quality of life (QOL) of patients,^[4]^ and about 13% of SCI patients die of urinary system complications.^[3]^ Although the short- and long-term efficacy and safety of urethral catheterization and surgical treatment for patients with NB have been confirmed,^[5,6]^ invasive procedures often lead to complications such as urinary system infection or urethral injury. Therefore, seeking a safer and more effective treatment for NB is the research focus and problem of clinical medical staff.

In recent years, functional magnetic stimulation (FMS) has become a hot spot in the treatment of NB. FMS, as a noninvasive treatment with accurate location, simple operation, comfort, and painlessness, has been popularized in clinics, showing the effect of improving patients’ urinary symptoms and bladder function.^[7,8]^ At present, the clinical research on the application of sacral nerve FMS to treat NB after SCI is increasing, but most of the published related research samples are small, and it is still controversial whether the curative effect of conventional therapy combined with sacral nerve FMS is better than conventional therapy. Therefore, this study systematically evaluates the additional therapeutic effect of basic therapy combined with sacral nerve magnetic stimulation on patients with NB after SCI, aiming at providing a reference and basis for clinical treatment of NB.

## 2. Materials and methods

The International Prospective Register of Systematic Reviews recorded this work (CRD42023481788). We write this article based on the Preferred Reporting Items for Systematic Reviews and Meta-Analyses guide.

### 2.1. Search method

We searched 4 English and 4 Chinese electronic databases: PubMed, Web of Science, Embase, Cochrane Library, China National Knowledge Infrastructure, VIP Database, China Biomedical Literature Database, and WanFang Data. We collected the randomized controlled trial (RCT) of NB after sacral nerve FMS treatment of SCI. The search time is limited from the establishment of the database to September 25, 2023. We adopt the search strategy of combining subject words with free words and make adjustments according to the search requirements of electronic databases. We first searched the PubMed database and then randomly searched other databases. The search strategy of the PubMed database is shown in Table 1.

**Table 1:** Search strategy.

Number	Search terms
#01	Spinal Cord Injuries [Mesh]
#02	(((((Spinal Cord Trauma [Title/Abstract]) AND (Myelopathy, Traumatic [Title/Abstract])) AND (Spinal Cord Transection [Title/Abstract])) AND (Spinal Cord Laceration [Title/Abstract])) AND (Post-Traumatic Myelopathy [Title/Abstract])) AND (Spinal Cord Contusion [Title/Abstract])
#03	#01 or #02
#04	Urinary Bladder, Neurogenic [Mesh]
#05	(((((((Neurogenic Urinary Bladder [Title/Abstract]) AND (Neurogenic Bladder [Title/Abstract])) AND (Urinary Bladder Neurogenic Dysfunction [Title/Abstract])) AND (Neurogenic Dysfunction of the Urinary Bladder [Title/Abstract])) AND (Neurogenic Urinary Bladder Disorder [Title/Abstract])) AND (Neuropathic Bladder [Title/Abstract])) AND (Neurogenic Bladder Disorders [Title/Abstract])) AND (Spastic Neurogenic Bladder [Title/Abstract])
#06	#04 or #05
#07	Magnetic Field Therapy [Mesh]
#08	(Electric Stimulation Therapy [Title/Abstract]) AND (Magnets [Title/Abstract])Randomized
#09	#07 or #08
#10	Nervi sacrales
#11	Randomized Controlled Trials [Mesh]
#12	((Clinical Trials, Randomized [Title/Abstract]) AND (Trials, Randomized Clinical [Title/Abstract])) AND (Controlled Clinical Trials, Randomized [Title/Abstract])
#13	#11 or #12
#14	#03 and #06 and #09 and #10 and #13

### 2.2. Inclusion and exclusion criteria

#### 2.2.1. Inclusion criteria

Research type: RCT. Subjects: patients with NB after SCI, regardless of their age, race, and nationality. Intervention measures: the control group was treated with basic treatment (the basic treatment may be different among different studies, and the basic treatment of the 2 groups in the same study must be the same), while the experimental group was treated with sacral nerve FMS on the basis of the control group. Outcome indicators: maximum bladder capacity, residual urine volume, bladder capacity at initial urination, QOL score, urination times, maximum urine volume, and single urine volume.

#### 2.2.2. Exclusion criteria

Review, comments, case reports, animal experiments, or repeated published literature; retrospective study; intervention measures combined with transcranial magnetic stimulation; unable to extract data or unable to carry out quantitative synthesis research based on existing data; and this study focuses on the study of outcome indicators.

### 2.3. Data extraction

Two researchers independently screened the literature, extracted the data, and cross-checked. In cases of disagreement, the third party assisted in judgment. The process of literature screening is to first browse the topics and abstracts and, after excluding obviously irrelevant studies, read through the full text to determine whether to include them in the analysis. The main information extracted includes the following: the basic information included in the study, such as the name of the first author, the year of publication, the title; baseline information of the subjects, such as sample size of each group, sex, age, and course of disease of patients; specific details of intervention measures, such as FMS frequency (Hz), sacral nerve site, intervention duration, frequency, and course of treatment; 7 elements of bias risk assessment; and outcome indicators of concern.

### 2.4. Risk assessment of bias included in the study

According to the bias risk assessment methods and tools of RCT in the Cochrane manual, 2 researchers conducted bias risk assessments on the included studies. To ensure accuracy, 2 researchers independently analyzed the following 6 aspects. Six aspects of the risk of bias are assessed: selection bias, implementation bias, measurement bias, follow-up bias, reporting bias, and other biases. We classified the risk of bias as low, high, or unclear. If there is any dispute, please ask the third researcher for assistance.

### 2.5. Statistical analysis

We used Stata software (version 15.0, Stata Corp, College Station, TX, USA) for meta-analysis. The measurement data adopt the mean difference (MD) and its 95% confidence interval (CI) as the effect quantity. The Q test and I^2^ test are used to test the heterogeneity among the included research results. If *P* > .10 and I^2^ ≤ 50%, the fixed effect model is used. If *P* ≤ .10 and I^2^ > 50%, it shows that there is obvious heterogeneity among the studies. The sources of heterogeneity are analyzed by subgroup analysis or sensitivity analysis. After excluding the influence of obvious clinical heterogeneity, the random effect model is used for meta-analysis, and the test level is set to α = 0.05. The funnel diagram was used to test the publication bias.

## 3. Results

### 3.1. Document screening process and results

**Figure 1.**
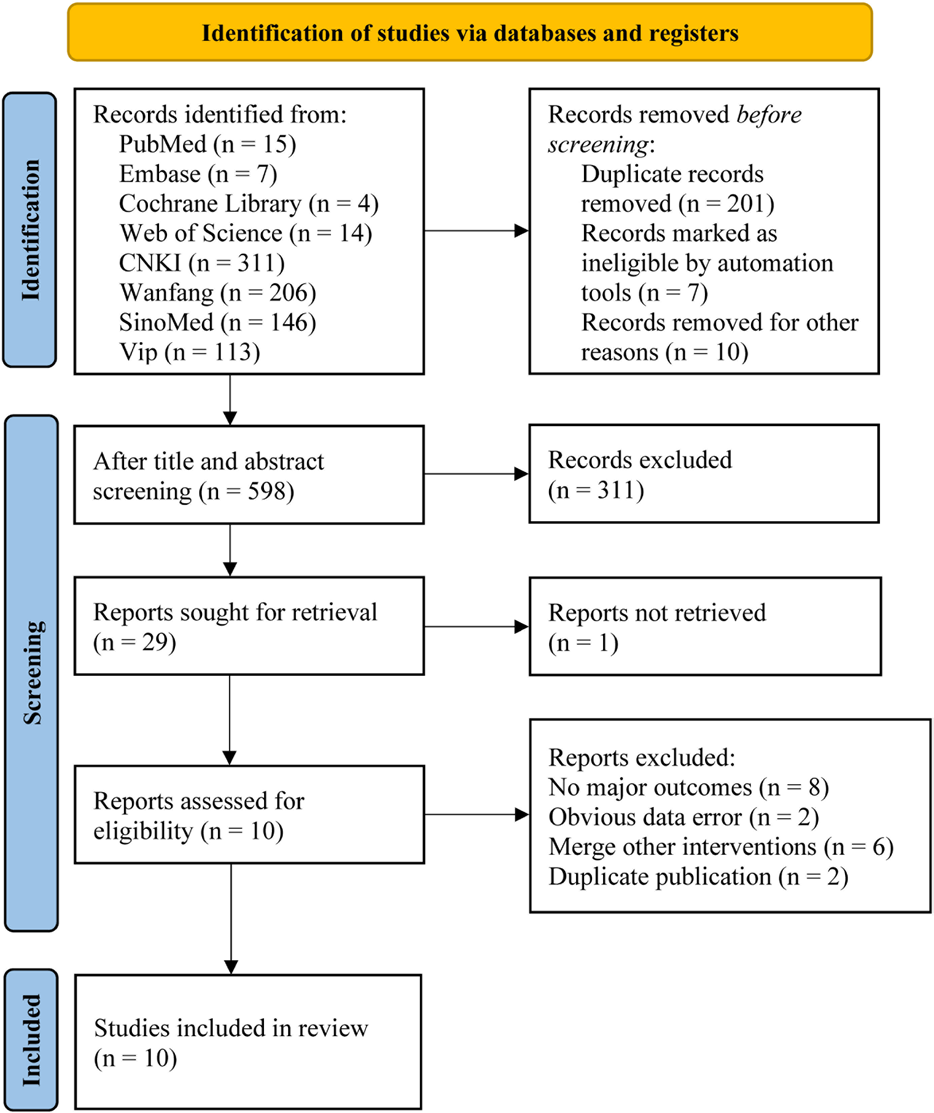
Flow diagram of studies identified.

According to the retrieval strategy, 818 related documents were obtained by preliminary retrieval. According to the exclusion criteria, 10 articles^[9–18]^ were finally obtained, involving 537 subjects. See Figure 1 for the process and results of literature screening.

### 3.2. Basic characteristics of inclusion in the study

The literature included is from 2015 to 2022, all of which are RCT studies published in China in recent years. The control group was given routine treatment, and the experimental group was combined with FMS on the basis of routine treatment. The shortest treatment time is 10 days, and the longest is 8 weeks. See Table 2 for the specific basic characteristics of the study.

**Table 2:** Characteristics of the included studies.

Author/year	Case number (T:C)	Gender (man/woman)	Age, yr	Course of a disease, mo	Intervention measure	Ganglion segment	Treatment time, wk	Outcome
Zhang 2021^[9]^	(15, 15:16)	9/6	48.27 ± 15.24	3 ± 1	(FMS [15 Hz] + UC, FMS [20 Hz] + UC):UC	S3	4	①②③④
		5/10	46.67 ± 11.68	2.93 ± 1.16				
		8/8	40.31 ± 13.17	2.38 ± 0.81				
Sun et al 2021^[10]^	(12, 13:13)				(FMS [5 Hz] + UC, FMS [10 Hz] + UC):UC	S1	10 days	①②
Song et al 2022^[11]^	(14, 15:15)	11/3	35.5 ± 9.85	2.54 ± 0.6	(FMS [5 Hz] + UC, FMS [20 Hz] + UC):UC	S3	4	①②
		11/4	36.27 ± 9.22	2.27 ± 0.64				
		13/2	34.47 ± 6.22	2.15 ± 0.75				
Ma et al 2020^[12]^	60:60	40/20	46.3 ± 9.5	5.7 ± 1.3	FMS (5 Hz) + UC:UC	S3	4	①
		38/22	45.8 ± 9.2	5.4 ± 1.2				
Hu et al 2021^[13]^	20:20	14/6	36.6 ± 9.16	6.3 ± 3.31	FMS (15 Hz) + UC:UC	S3	8	①②③④⑤⑥⑦
		12/8	34.75 ± 11.88	6.55 ± 2.11				
Cehn and Zhang 2022^[14]^	47:47	27/20	43.11 ± 4.98		FMS (15 Hz) + UC:UC	S3	8	①②③⑤⑥⑦
		25/22	43.13 ± 4.66					
Song et al 2019^[15]^	20:20	10/10	36.26 ± 12.08	7.22 ± 2.19	FMS (15 Hz) + UC:UC	S3	8	①②③④⑤⑥⑦
		12/8	37.29 ± 12.95	7.13 ± 2.38				
Li et al 2015^[16]^	20:20	10/10	38.25 ± 12.74	6.84 ± 2.08	FMS (10 Hz) + UC:UC	S3	4	①②③④⑤⑥⑦
		11/9	39.35 ± 13.66	6.76 ± 2.26				
Jiang 2022^[17]^	20:19	17/3	47.7 ± 9.3	3.6 ± 1	FMS (20 Hz) + UC	S3	8	②③④
		15/4	46.8 ± 8.4	3.5 ± 1	UC			
Han 2021^[18]^	18:18	9/9	52.0 ± 4.7		FMS (15 Hz) + UC:UC	S	4	①③
		10/8	53.0 ± 1.3					

①: maximum bladder capacity; ②: residual urine volume; ③: bladder capacity at first urination; ④: QOL score; ⑤: number of urinations; ⑥: maximum urine output; and ⑦: single urine volume.

C = control group, FMS = functional magnetic stimulation, Hz = hertz, QOL = quality of life, S = nervi sacrales, T = treatment group, UC = conventional therapy.

### 3.3. The results of biased risk assessment included in the study

**Figure 2.**
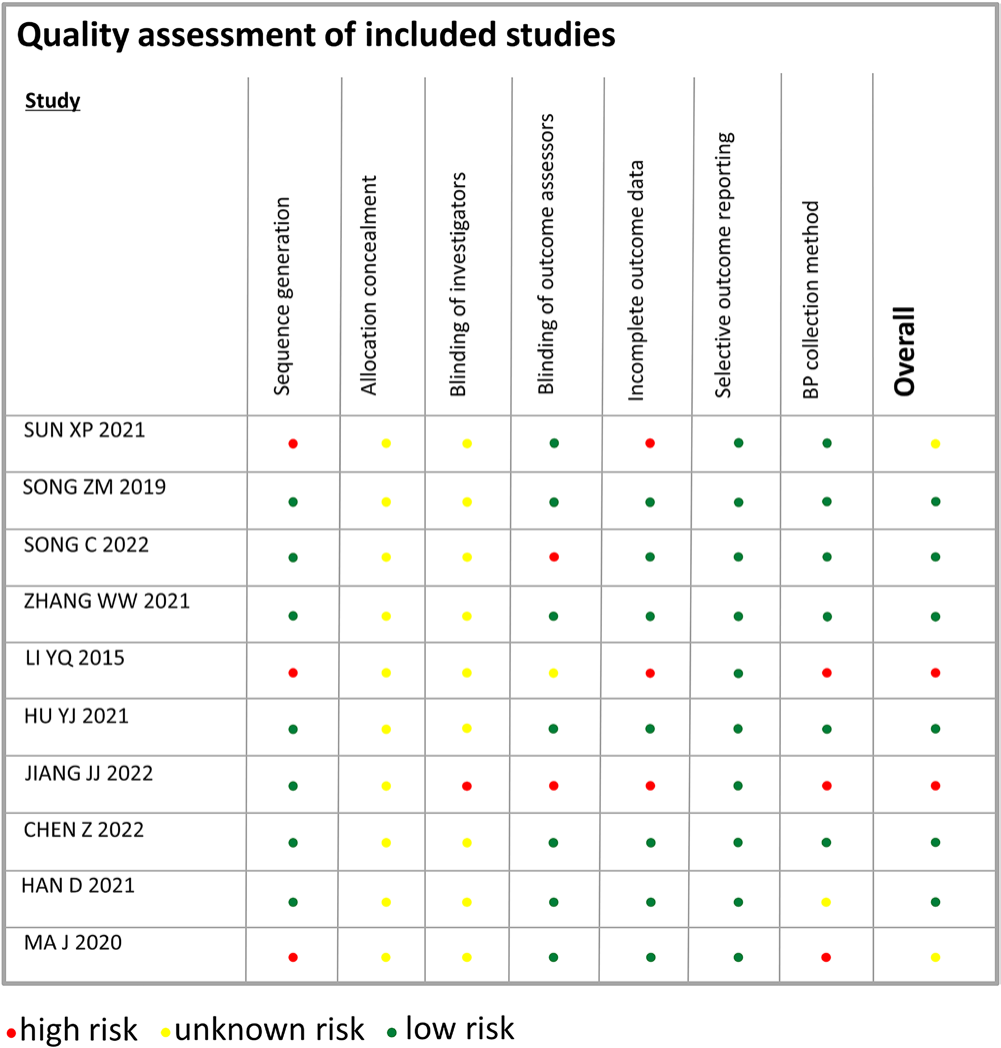
Risk of bias of the included studies.

The 10 articles included in the study all followed the principle of randomized control. Three papers^[10,12,16]^ did not specify random methods, so they were classified as high risk. Seven articles^[1,11,13–15,17,18]^ showed the randomized method and were rated as low risk. None of the 10 studies specified the distribution concealment, so they were rated as unknown risks. See Figure 2 for specific bias risk assessment results.

### 3.4. Meta-analysis results

#### 3.4.1. Effect of sacral nerve FMS on bladder volume measurement index

##### 3.4.1.1. Maximum bladder capacity

**Figure 3.**
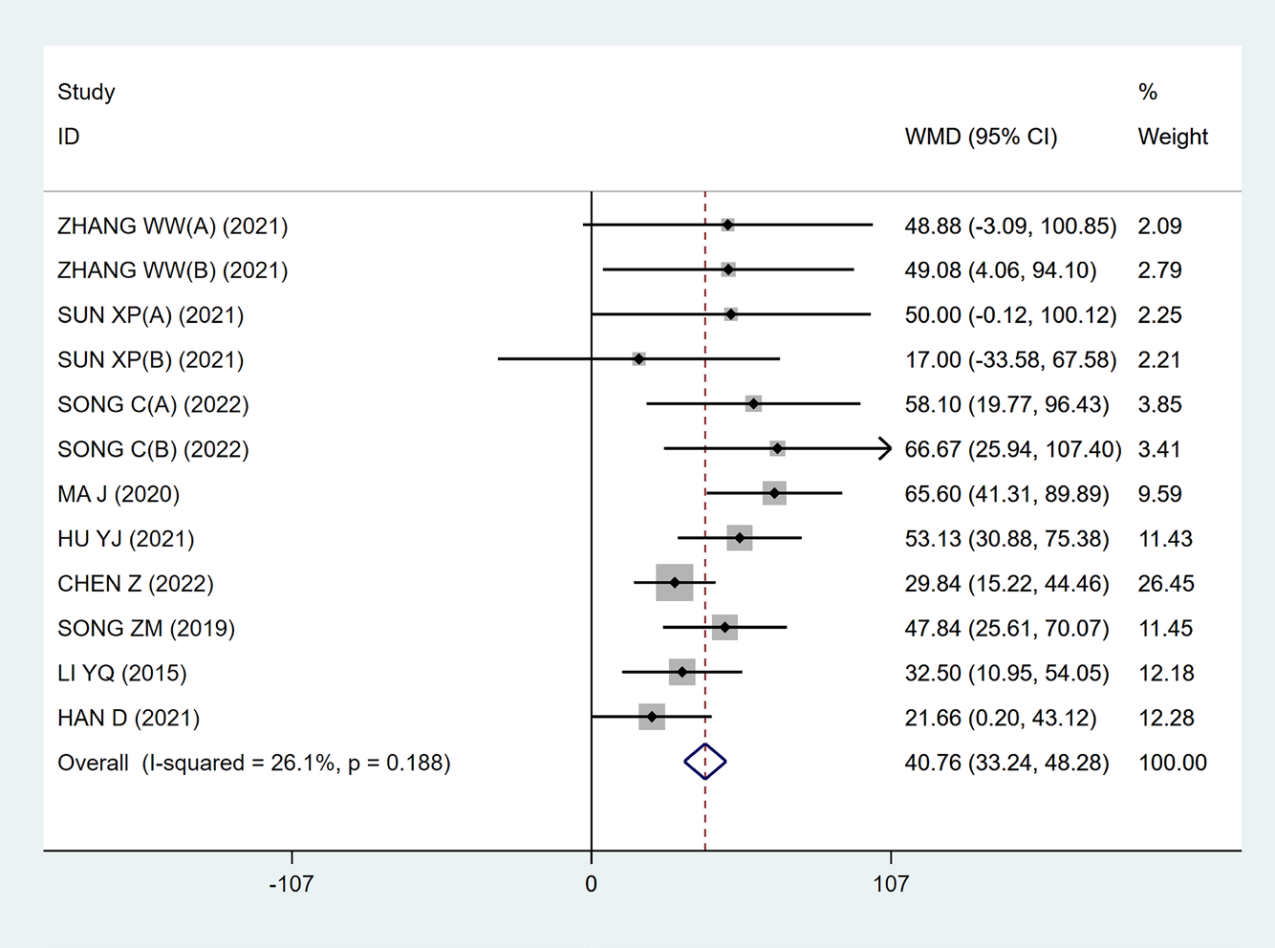
Forest plots of maximum bladder capacity. CI = confidence interval.

Nine studies^[9–16,18]^ analyzed the maximum bladder capacity, involving 497 patients. After the heterogeneity test, there was no heterogeneity between the studies (*P* = .188; I^2^ = 26.1%; Fig. 3), so the fixed effect model was selected. Finally, it is concluded that the experimental group can effectively expand the bladder capacity, and the difference is statistically significant (MD, 40.76 [95% CI, 33.24–48.28]; *P*˂.05; Fig. 3).

##### 3.4.1.2. Residual urine volume

**Figure 4.**
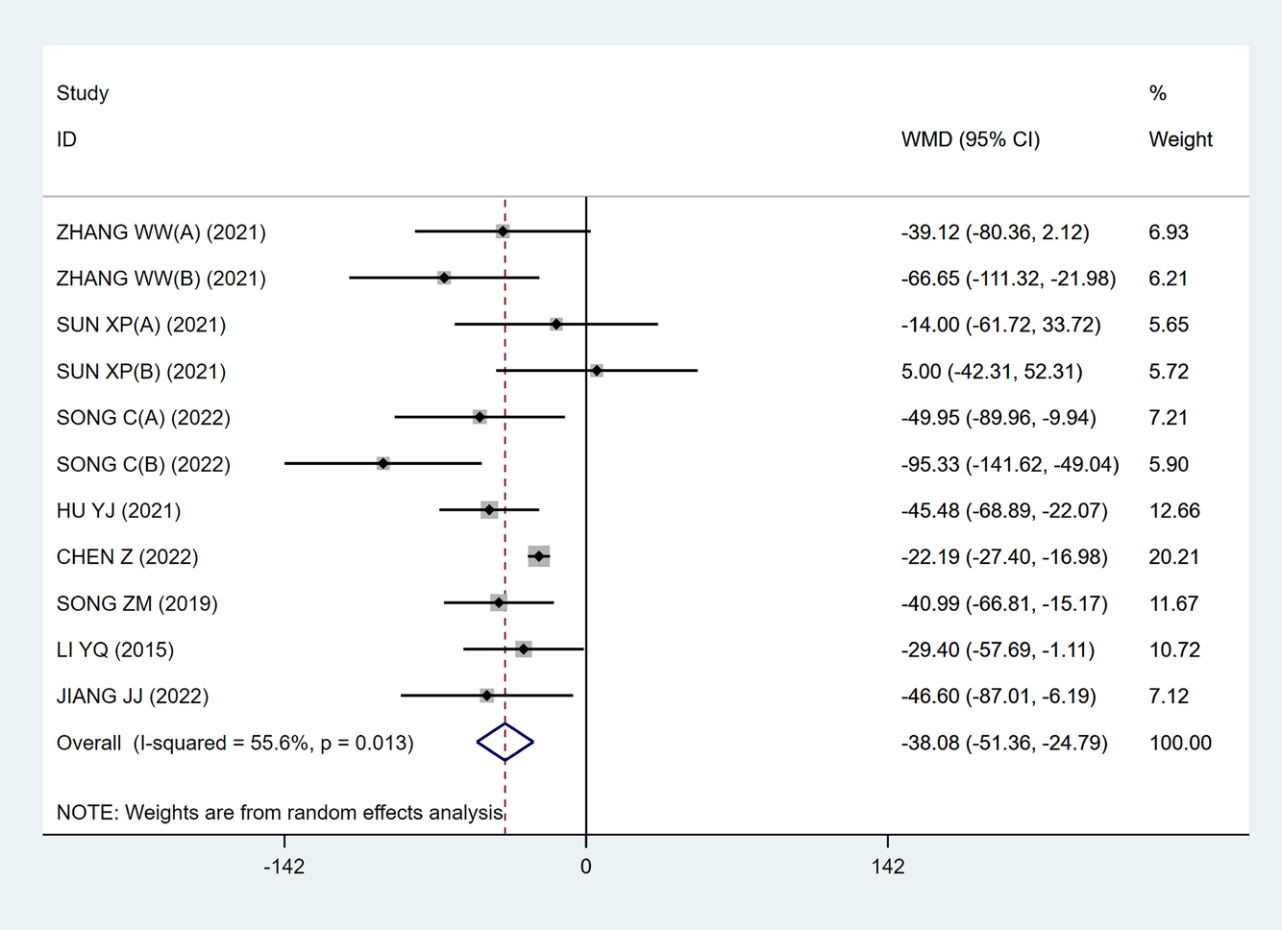
Forest plots of residual urine volume. CI = confidence interval.

**Figure 5.**
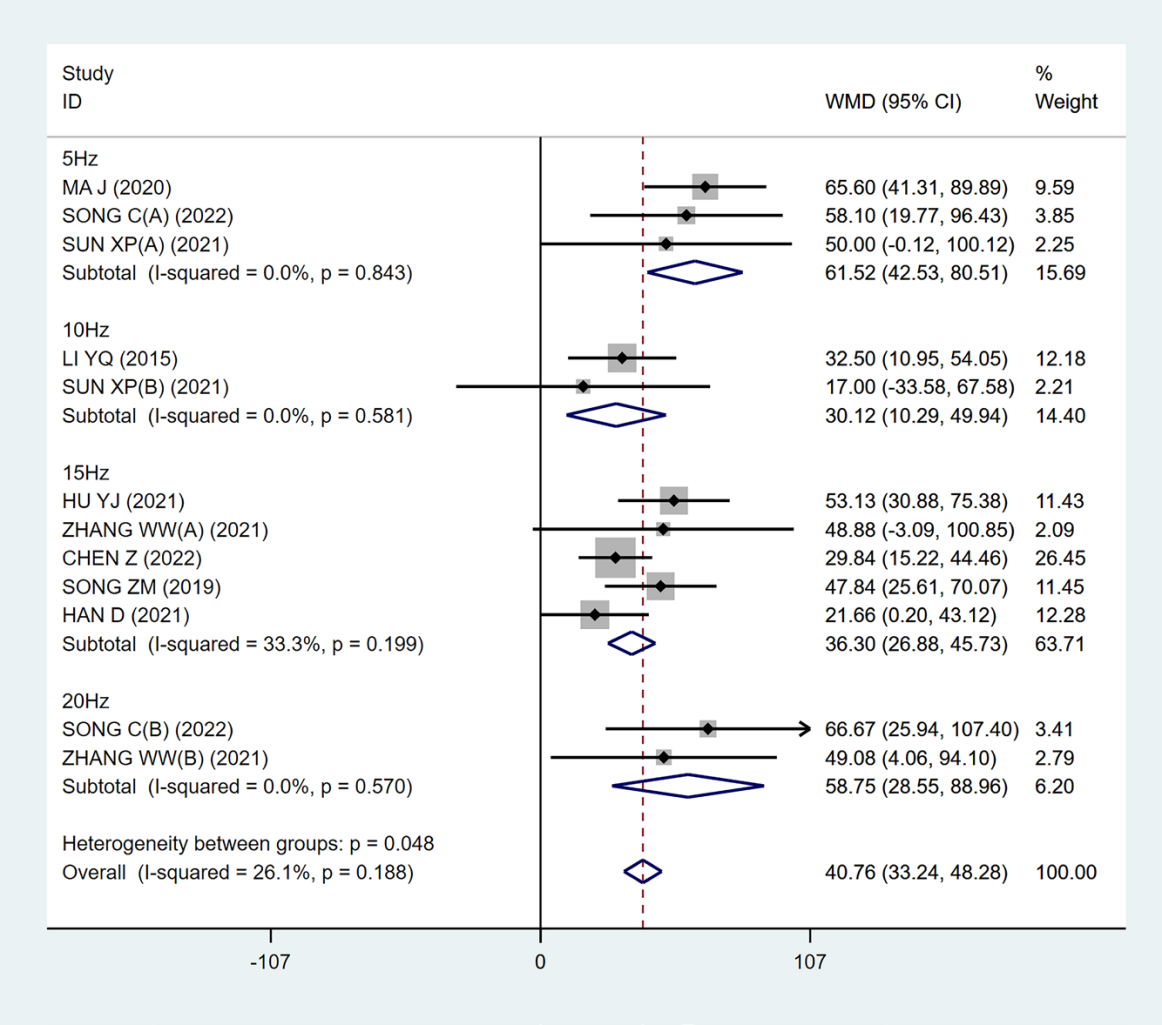
Forest plots of subgroup analysis of residual urine volume. CI = confidence interval.

Eight studies^[9–11,13–17]^ used residual urine volume to evaluate the therapeutic effect, including 380 subjects. The results showed MD, −38.08 (95% CI, −51.36 to −24.79; *P*˂.05; Fig. 4), which indicated that FMS treatment of sacral nerve was beneficial to reduce the residual urine volume of patients with NB after SCI. However, there is heterogeneity between studies (*P* = .013; I^2^ = 55.6%; Fig. 4). Subsequently, we conducted a sensitivity analysis on the indicators of residual urine volume and evaluated the influence of various studies on the summary results by one-by-one exclusion method. The analysis results show that after excluding the study of Chen and Zhang,^[14]^ the heterogeneity (I^2^) is reduced from 55.6% to 27.7% (Fig. 5). As shown in the figure, its heterogeneity may be affected by this study. Through reading the full text, it is found that an unreported course of disease and a longer course of treatment may be the source of its heterogeneity.

##### 3.4.1.3. Bladder capacity at the beginning of urination

**Figure 6.**
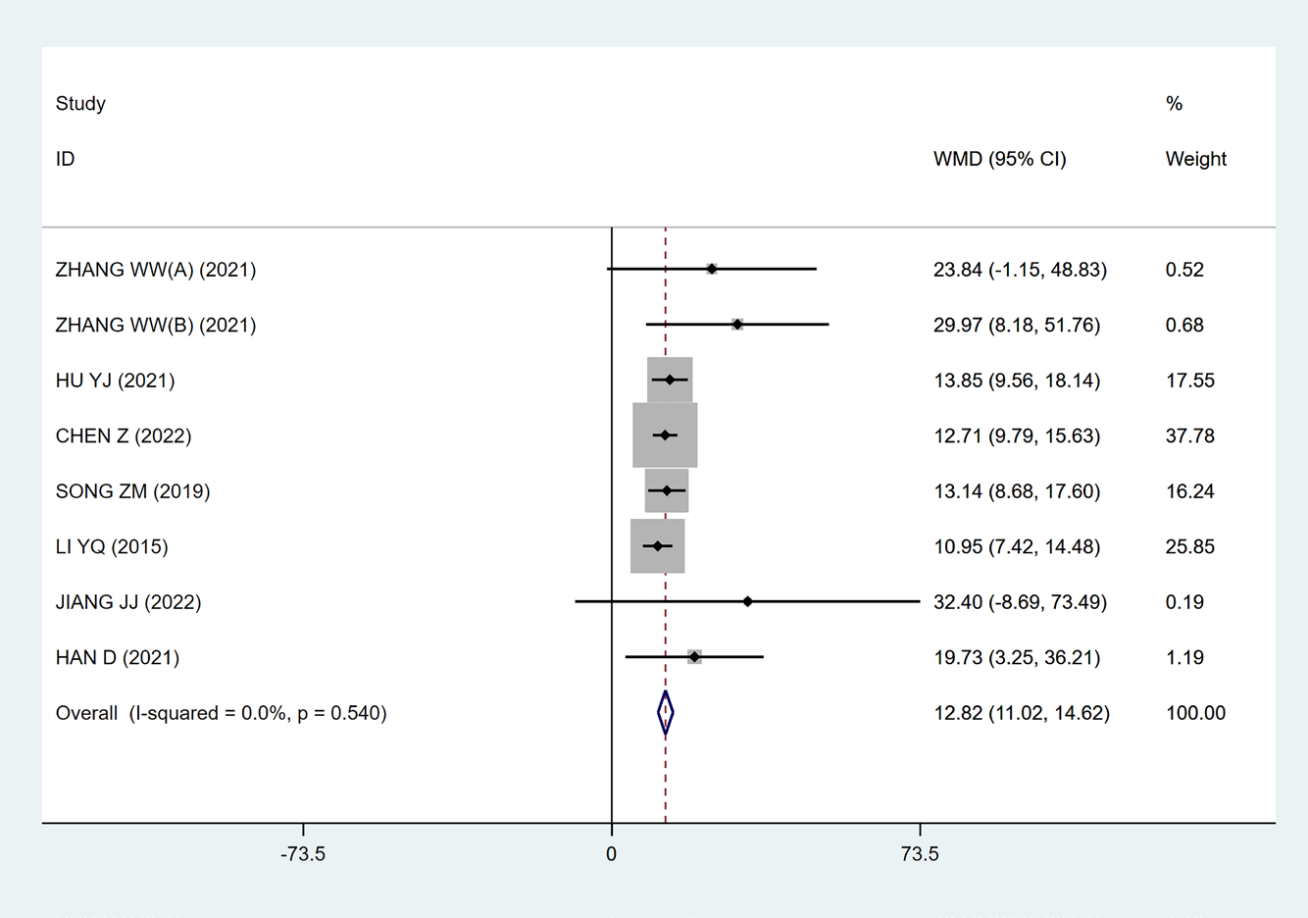
Forest plots of bladder capacity at the beginning of urination. CI = confidence interval.

Seven studies^[9,13–18]^ used bladder capacity at the beginning of urination as an outcome index, and the greater the bladder capacity, the more obvious the prompt effect. It was found that after FMS treatment of the sacral nerve, the bladder capacity of patients at the time of first urination was obviously improved, and there was no heterogeneity between the studies (*P* = .54; I^2^ = 0; Fig. 6), with statistical significance (MD, 12.82 [95% CI, 11.02–14.62]; *P*˂.05; Fig. 6).

#### 3.4.2. Effect of sacral nerve FMS on urination diary index

Four studies^[13–16]^ recorded 3 kinds of micturition diaries (single micturition, micturition times, and maximum micturition) to evaluate the curative effect, including 214 subjects, 107 in the experimental group, and 107 in the control group.

##### 3.4.2.1. Single urine volume

**Figure 7.**
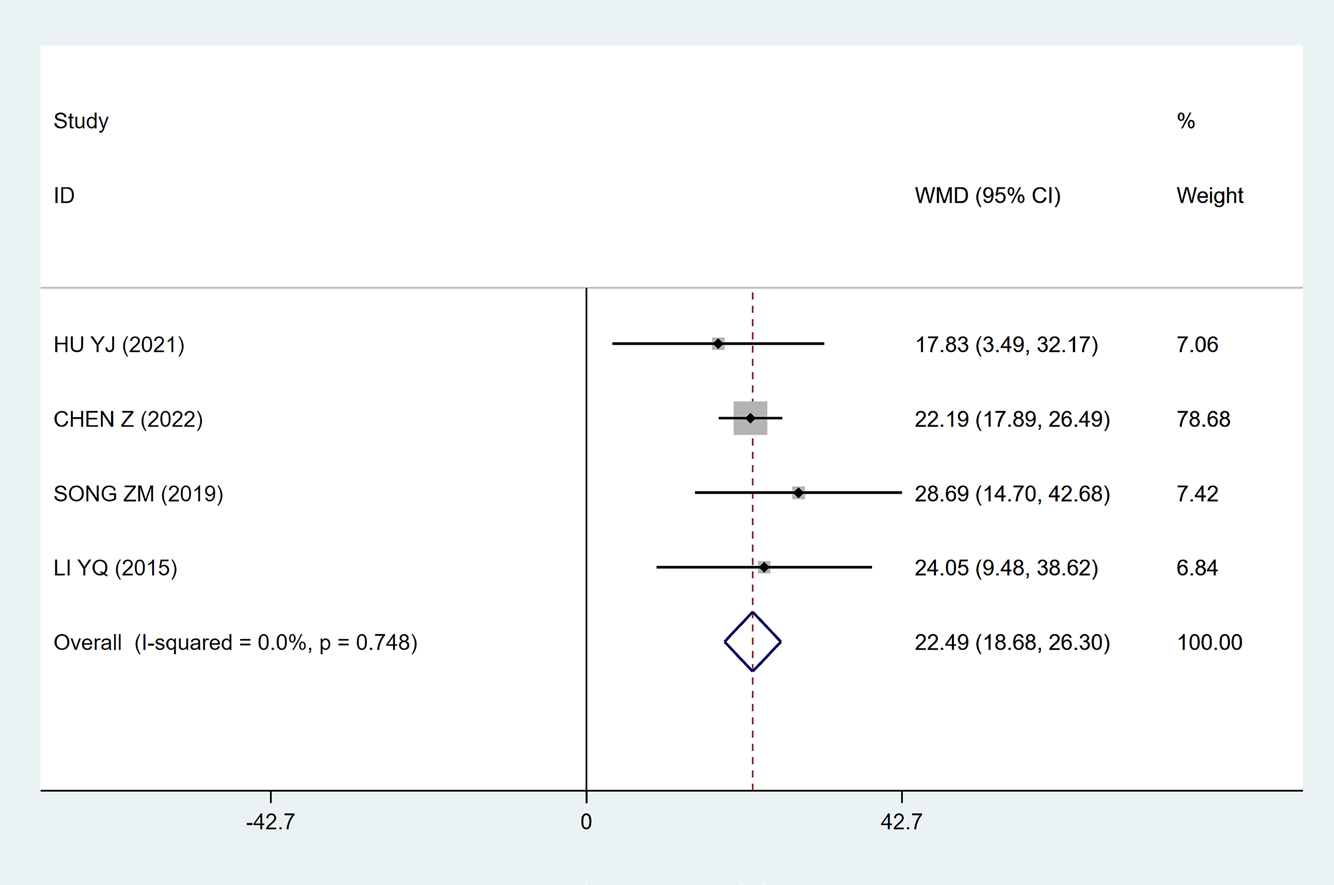
Forest plots of single urine volume. CI = confidence interval.

The more the single urine volume increases, the more significant the curative effect will be. It was found that the sacral nerve FMS group could increase the single urine volume compared with the control group, and the difference was statistically significant (MD, 22.49 [95% CI, 18.68–26.30); *P*˂.05; Fig. 7), and there was no heterogeneity among the studies (*P* = .748; I^2^ = 0; Fig. 7).

##### 3.4.2.2. The frequency of urination

**Figure 8.**
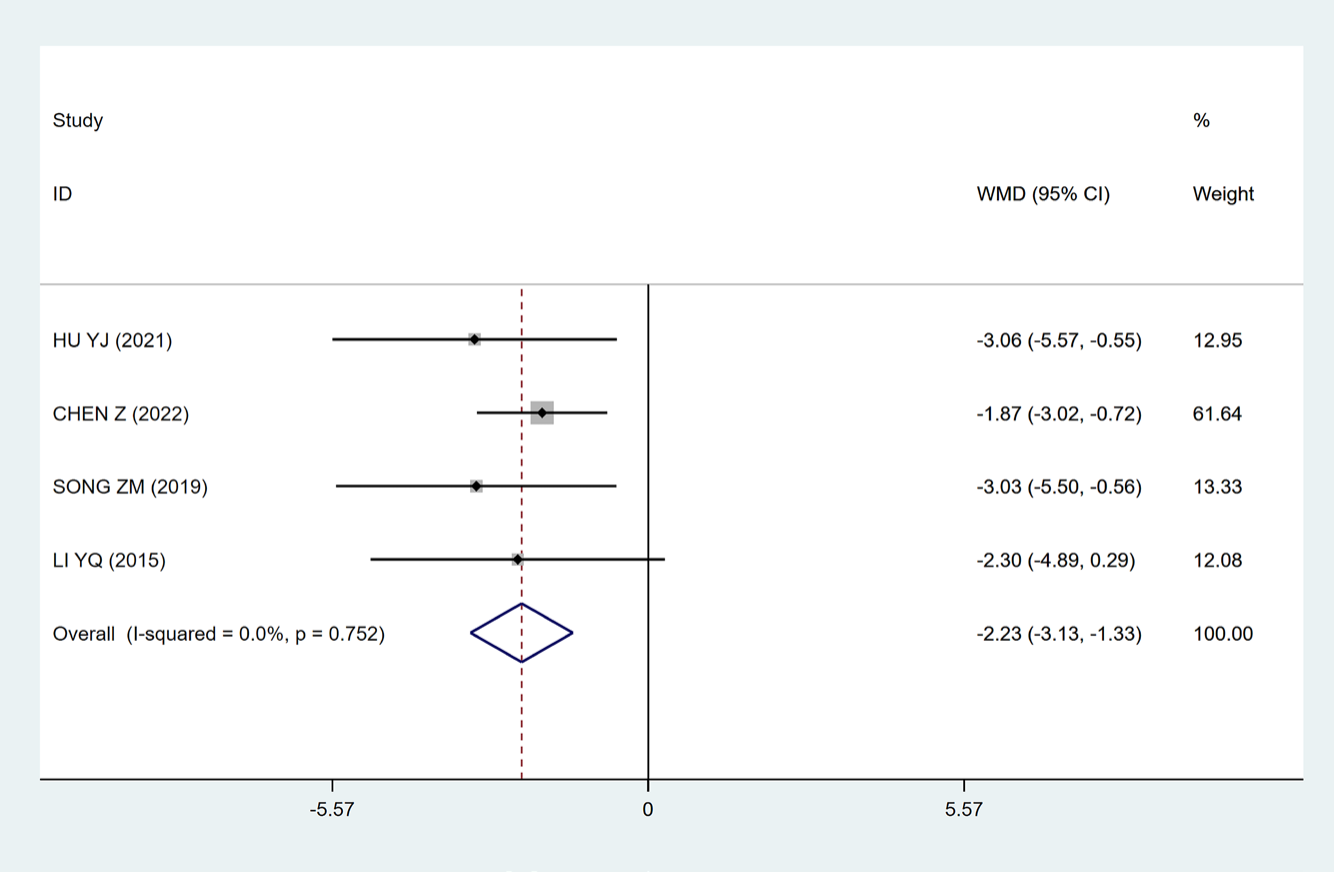
Forest plots of the frequency of urination. CI = confidence interval.

The decrease in the frequency of urination indicates that the treatment is effective. It was found that the sacral nerve FMS group could effectively urinate fewer times than the conventional treatment group, which indicated that the experimental group was superior to the control group, with statistical significance (MD, −2.23 [95% CI, −3.13 to −1.33] *P*˂.05; Fig. 8), and there was no heterogeneity among the studies (*P* = .752; I^2^ = 0; Fig. 8).

##### 3.4.2.3. Maximum micturition

**Figure 9.**
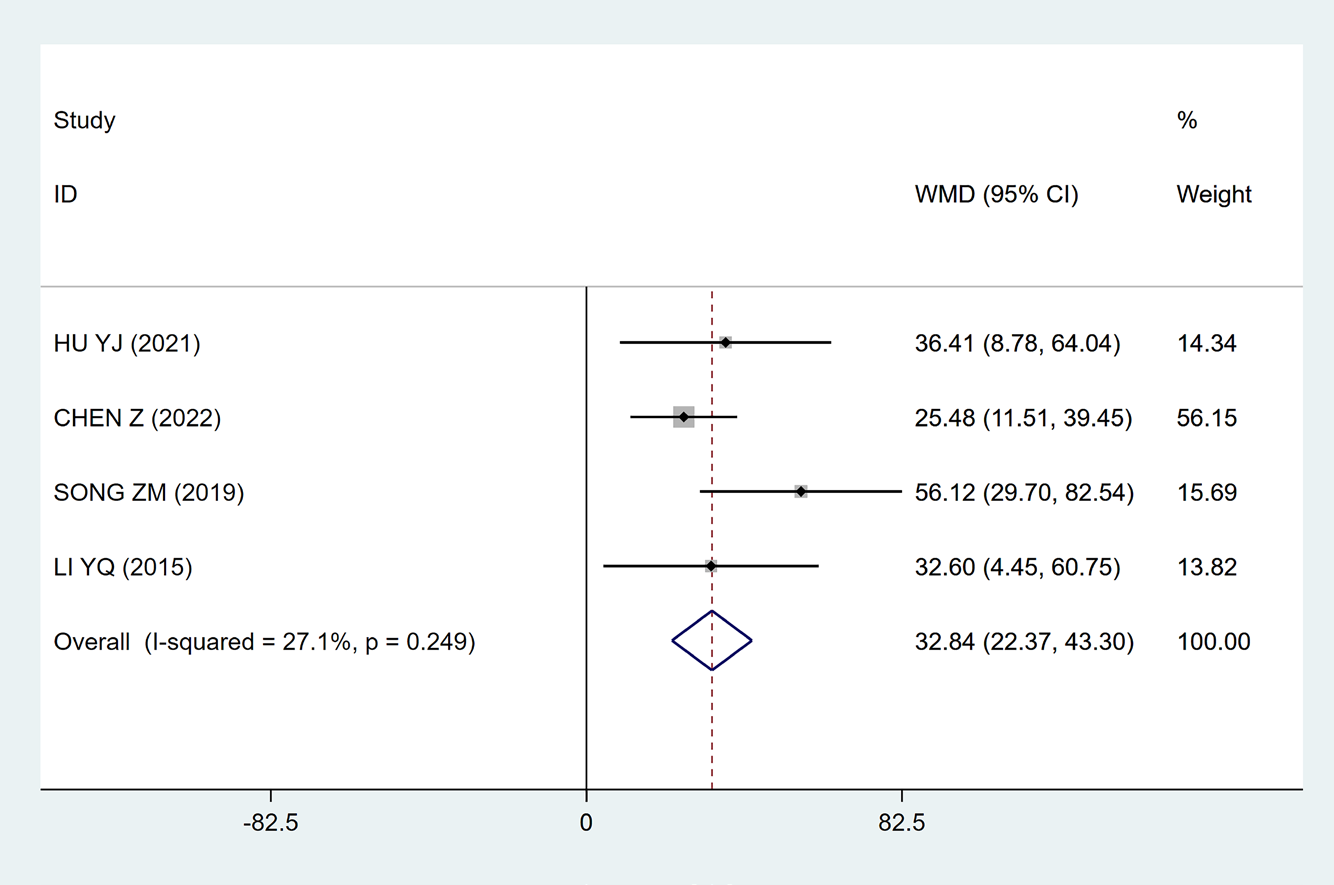
Forest plots of maximum micturition. CI = confidence interval.

The greater the maximum micturition, the more obvious the curative effect. Because there is no heterogeneity between studies (*P* = .249; I^2^ = 27.1%; Fig. 9), the fixed effect model is adopted. Meta-analysis of the fixed effect model showed that the sacral nerve FMS group could increase the maximum urine output compared with the control group, and the difference was statistically significant (MD, 32.84 [95% CI, 22.37–43.30]; *P*˂.05; Fig. 9).

#### 3.4.3. Effect of sacral nerve FMS on QOL score

**Figure 10.**
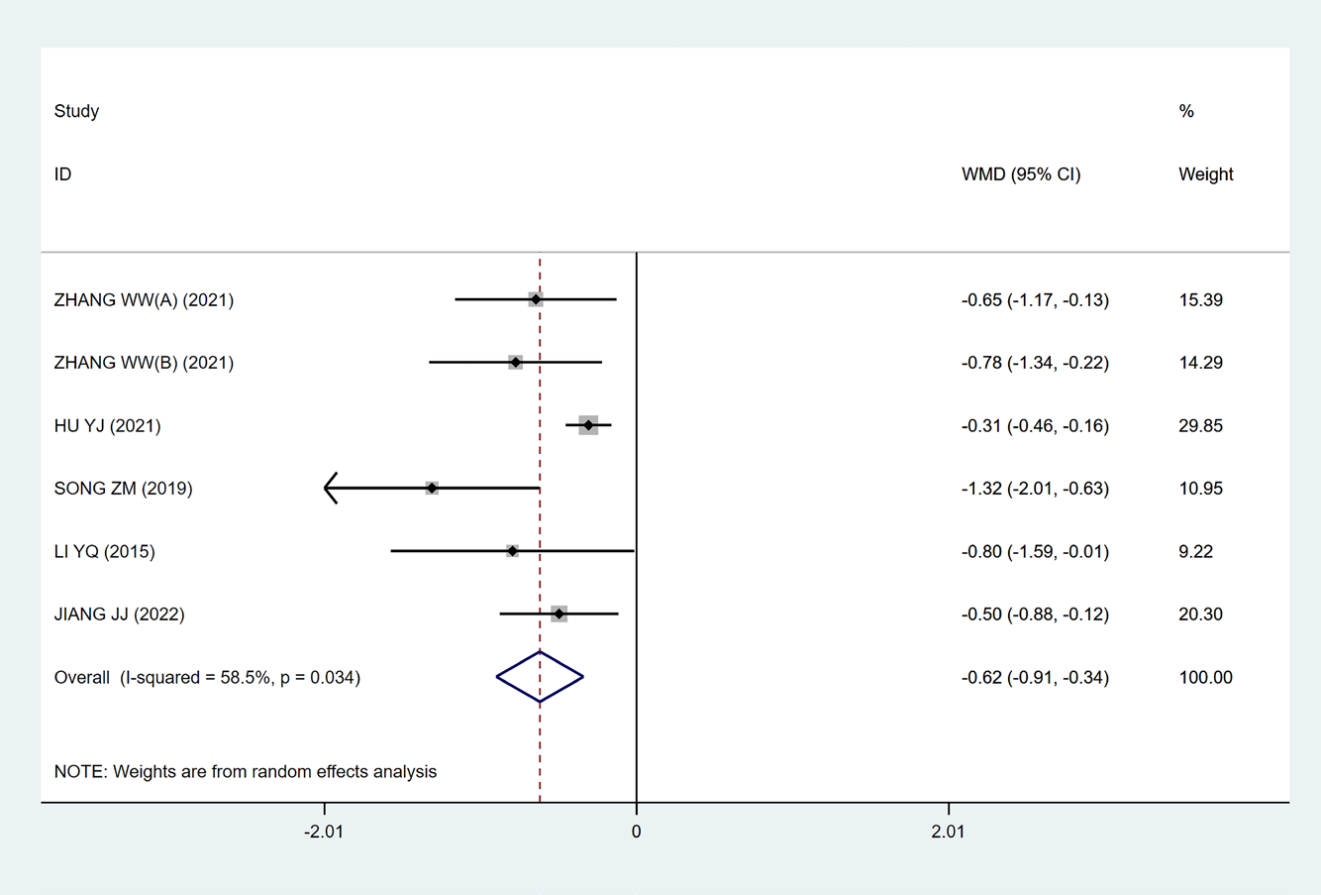
Forest plots of the quality-of-life score. CI = confidence interval.

Five studies^[9,13,15–17]^ evaluated the QOL score at the end point of treatment, including 204 subjects. The decrease in QOL score shows that a good curative effect has been achieved. Because of the heterogeneity among the studies (*P* = .034; I^2^ = 58.5%; Fig. 10), the random effect model was adopted. The results showed that the sacral nerve FMS group could lower the QOL score and improve the QOL to some extent compared with the control group, and the difference was statistically significant (MD, −0.62 [95% CI, −0.91 to −0.34; *P*˂.05; Fig. 10).

#### 3.4.4. Sensitivity analysis

The sensitivity analysis of 7 indicators in this meta-analysis was carried out by excluding individual studies one by one. After requantitative synthesis, no obvious changes were found in the above research results, suggesting that the research results are more stable.

### 3.5. Publication bias analysis

**Figure 11.**
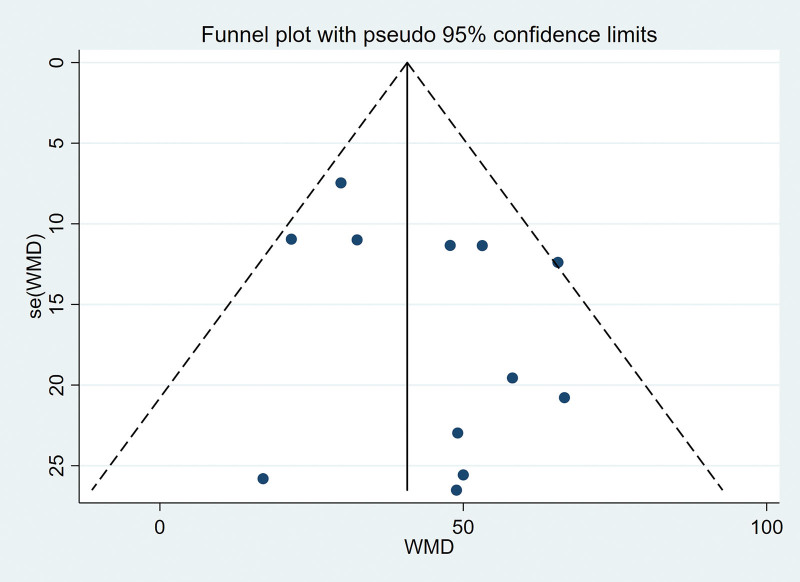
Funnel plot of the publication bias.

The maximum bladder capacity was selected as the outcome index, and the funnel chart was drawn to test whether there was publication bias. The distribution of each research point in the figure lacks corresponding symmetry, suggesting that there may be the possibility of publication bias (Fig. 11).

## 4. Discussion

The results of this meta-analysis show that for patients with NB after SCI, the combination of conventional treatment and sacral nerve magnetic stimulation can effectively increase the maximum bladder capacity and the bladder capacity at the beginning of urination and reduce the residual urine volume. It has an obvious curative effect on the urination diary: the single urine volume and maximum urine volume of patients increase, and the number of urinations decreases. Applying this treatment method to treat patients can effectively reduce their QOL score, thus improving life therapy.

The results of this study support the curative effect of sacral nerve magnetic stimulation on patients with NB after SCI, and it is mainly considered that the following 4 aspects have played a key role^[19]^: inhibiting the excessive activity and excitement of spastic bladder detrusor; increase the maximum urethral closure pressure, increase the urine storage capacity of bladder, and prolong the urine storage period; promoting detrusor contraction; and improve the function of pelvic floor muscles. In addition, some studies believe that FMS can regulate nerve activity and promote the dynamic balance of various nerve functions.^[20]^ However, the exact mechanism of FMS in treating patients with NB after SCI is still unclear, which will be the focus of future research.

The positive results of this meta-analysis are consistent with most published related research results.^[9–18,21–23]^ It should be noted that there are nonsingle factors among the reported related research groups, such as the experimental group combined with sacral nerve FMS and repetitive transcranial FMS on the basis of the control group,^[21]^ combined with sacral nerve FMS and other drugs,^[22]^ or combined with sacral nerve FMS and acupuncture.^[23]^ These clinical studies of combined therapy cannot provide strict evidence supporting the effectiveness of sacral nerve FMS. In order to further clarify the curative effect of sacral nerve FMS on patients with NB after SCI, this study conducted a systematic evaluation and meta-analysis with a more rigorous scheme. The RCT control group included in the analysis was given basic treatment, and the experimental group was given only sacral nerve FMS treatment on the basis of the control group. It is worth noting that the basic treatment among the studies may not be completely the same, but the basic treatment between the 2 groups in the same study must be the same. Based on this premise, the differences between the RCT groups included can be explained by sacral nerve FMS. The RCT included in this study is mainly based on the literature published in the last 3 years. It is the first time to report a meta-analysis on this issue at home and abroad, which has certain reference significance for the clinical treatment of NB after SCI.

There are still some limitations in this study: the metalimitation of this meta-analysis is the low methodological quality of the included studies; all the included studies are Chinese literature, and all of them have reported positive results, so publication bias cannot be avoided^[24]^; the basic treatment of each study is different, which may have some influence on the differences between groups and become a potential bias in this study; and because the treatment of patients with NB with SCI by FMS is still in the exploratory stage, there is still a big difference in the stimulation frequency of existing studies, but the stimulation frequency plays a key role in the treatment effect. Unfortunately, this meta-analysis cannot give the optimal range of magnetic stimulation frequency based on the existing evidence for the time being, and it will be further analyzed when more related studies are included in the future.

## 5. Conclusion

The existing evidence shows that the combination of conventional treatment and FMS of the sacral nerve is superior to conventional treatment in improving the clinical symptoms of patients with NB after SCI. However, due to the limitation of the quality and quantity of included studies, the above conclusions should be carefully interpreted, and more high-quality related studies are needed to verify them.

## Acknowledgments

Research on medication rules and clinical experience of Professor LiuBailing, a master of traditional Chinese medicine based on data mining (subject of Jilin Provincial Administration of Traditional Chinese Medicine; No. 2022153).

## Author contributions

**Conceptualization:** Xingxing Yu, Jianye Chen.

**Writing – original draft:** Xingxing Yu.

**Data curation:** Jianye Chen, Mingda Liu.

**Formal analysis:** Jianye Chen, Yunfei Li.

**Investigation:** Mingda Liu, Yunfei Li.

**Software:** Mingda Liu, Yunfei Li.

**Methodology:** Yuyan Jia, Hongsheng Zhan.

**Writing – review & editing:** Xukai Wang.
